# Analysis of therapeutic effect and safety of target-dose metoprolol in the treatment of patients with diabetes mellitus with chronic heart failure

**DOI:** 10.12669/pjms.301.3908

**Published:** 2014

**Authors:** Xuyang Liu, Chengfu Zhong, Pengtai Zhao, Zhihua Zhang, Ning Jia, Sheng’ou Su, Benliang Zou, Yuming Song

**Affiliations:** 1 Xuyang Liu, Department of Endocrinology, 252 Hospital of PLA, Baoding 071000, P. R. China.; 2 Chengfu Zhong, Department of Endocrinology, 252 Hospital of PLA, Baoding 071000, P. R. China.; 3 Pengtai Zhao, Department of Endocrinology, 252 Hospital of PLA, Baoding 071000, P. R. China.; 4 Zhihua Zhang, Department of Endocrinology, 252 Hospital of PLA, Baoding 071000, P. R. China.; 5 Ning Jia, Department of Endocrinology, 252 Hospital of PLA, Baoding 071000, P. R. China.; 6 Sheng’ou Su, Department of Endocrinology, Second Hospital of Hebei Medical University, Shijiazhuang 050000, P. R. China.; 7 Benliang Zou, Department of Endocrinology, Xiyuan Hospital CACMS, Beijing 010000, P. R. China.; 8 Yuming Song, Department of Traditional Chinese Medicine, First Center Hospital of Baoding, Baoding 071000, P. R. China.

**Keywords:** Chronic heart failure, Diabetes mellitus, Metoprolol, Therapeutic effect, Safety

## Abstract

***Objective: ***To explore the therapeutic effect and safety of target-dose metoprolol in treating chronic heart failure (CHF) patients complicated with diabetes mellitus (DM).

***Method*** ***s*** ***: ***One hundred and fifty-four elderly patients were randomly divided into an observation group and a control group (n=77), which were treated with target-dose metoprolol and conventional therapy, and routinely treated respectively. The New York Heart Association (NYHA) classification, left ventricular end-systolic diameter (LVESD), left ventricular end-diastolic diameter (LVEDD), left ventricular ejection fraction (LVEF), 6-min walking distance and medication safety of the two groups were compared.

***Results: ***Compared with the results before treatment, the NYHA classification, LVESD, LVEDD, LVEF and 6-minutes walking distance of both groups were significantly improved (P<0.05), with significantly better results in the observation group than those in the control group after treatment (P<0.05). In the 6 months of follow-up, the incidence of cardiac events in the observation group (3.90%) was significantly lower than that of the control group (14.29%) (P<0.05). The levels of average fasting blood sugar and glycosylated hemoglobin in the groups showed no significant differences (P>0.05).

***Conclusion: ***Treating CHF patients complicated with DM with target-dose metoprolol can obviously boost the cardiac function and exercise tolerance, leading to satisfactory clinical therapeutic effect, high security and moderate tolerance.

## INTRODUCTION

As the proportion of elderly in the population rises, chronic heart failure is becoming increasingly prevalent,^1^ leading to unacceptably high mortality rates worldwide^.^^2^ Metoprolol, as a β-receptor blocker, has been widely used in the clinical treatment of angina, hypertension, hypertrophic cardiomyopathy, arrhythmia, aortic dissection and hyperthyroidism, etc.^3^ Besides, it is able to remarkably decrease the mortality rate of heart failure patients^4^ by improving cardiac functions, left-ventricular remodeling and physical exercise-related capacity.^5^ Although metoprolol may delay the diagnosis of diabetes mellitus (DM) by counteracting hypoglycemia symptoms such as palpitation, it remains advantageous for DM patients.^6^^,^^7^

Heart failure, which refers to series of clinical signs and symptoms induced by reduced myocardial contractility and cardiac output, is clinically manifested as asthenia, dyspnea, fluid retention, vomiting, and swelling pain in hepatic region, etc., with relatively low 5-year survival rates.^8^^-^^10^ Effective treatment protocols should be able to improve the quality of life and to relieve relevant symptoms in the short-term, as well as to minimize myocardial remodeling, to prevent complications, and to decrease the recurrence and mortality rates of patients in the long-term. Meanwhile, rising blood sugar level can dramatically elevate the incidence rates of heart failure and other cardiovascular events.^11^^-^^13^ Metoprolol can bring about satisfactory clinical outcomes for chronic heart failure patients complicated with DM by blocking sympathetic nervous system. As to DM patients without heart failure, β-receptor blocker remains preventive.^14^ It has previously been reported that β-receptor blocker carvedilol was able to lower the mortality rates of myocardial infarction patients as a subgroup of DM cases by reversing myocardial remodeling and suppressing the hyperactivation of sympathetic nervous system.^15^ Compared with the therapeutic effects of low-dose metoprolol, high doses managed to enhance the cardiac functions and LVEF of patients, indicating that to tolerated dose or maximum medication dose is prerequisite for treating chronic heart failure patients complicated with DM.

Elderly heart failure patients complicated with DM receiving treatment from January 2010 to December 2012 were enrolled, aiming to clarify the clinical therapeutic effect and security of target-dose metoprolol.

## METHODS

***General*** ***I*** ***nformation: ***Elderly heart failure patients complicated with DM receiving treatment in our hospital from January 2010 to December 2012 were enrolled as the subjects. Inclusion criteria: 1) aged 60-85 years old; 2) Grade II, III or IV according to New York Heart Association (NYHA) classification; 3) non-insulin-dependent DM (type II DM) for 2 years or longer, with controllable fasting and postprandial blood sugar levels; 4) left ventricular ejection fraction (LVEF) lower than 40%; 5) good medication compliance; 6) adaptable to follow-up. Exclusion criteria: 1) acute coronary syndrome patients complicated with active myocarditis and heart failure; 2) uncontrollable hypertrophic cardiomyopathy or valvular heart disease; 3) uncontrollable complicated atrioventricular block (>Degree 2), sick sinus syndrome and other arrhythmia diseases; 4) recurrence of lung diseases such as chronic obstructive pulmonary disease; 5) heart rate<55 bpm, diastolic pressure>110 mmHg, systolic pressure>180 mmHg or <85 mmHg; 6) severe insufficiency of hepatic and renal functions; 7) with the history of adverse reactions after taking metoprolol; 8) inadaptable to medication and follow-up.

***Treatment*** ***P*** ***rotocol: ***The control group were routinely treated with hypoglycemics (e.g. acarbose) + diuretics (e.g. spironolactone) + vasodilators (e.g. isosorbide mononitrate) + digoxin + angiotensin-converting enzyme inhibitors (e.g. captopril). The doses were determined based on the clinical symptoms, signs, disease development as well as hepatic and renal functions.

In addition to the above medication, the observation group were simultaneously treated with β-receptor blocker metoprolol at the initial dose of 6.25 mg po bid, which was increased every other week to 100 mg po bid or to the tolerated maximum.

***Observation*** ***I*** ***ndices of*** ***T*** ***herapeutic*** ***E*** ***ffect and*** ***S*** ***ecurity: ***All the 154 patients were followed up for 6 months. The NYHA classification, left ventricular end-systolic diameter (LVESD), left ventricular end-diastolic diameter (LVEDD), LVEF and 6-min walking distance of the two groups were compared. The levels of blood lipid and blood sugar, resting heart rate, hepatic and renal functions, edema and blood pressure were monitored, and cardiac events such as arrhythmia, sudden death and aggravated heart failure were recorded.

***Statistical*** ***A*** ***nalysis: ***All the collected data were analyzed by SPSS 17.0. The measurement data were expressed as x±s and compared by the t test. The numeration data were expressed as n and compared by the χ^2^ test. P<0.05 was considered as statistically significant difference.

## RESULTS

A total of 154 cases were enrolled in this study according to above criteria, including 98 males and 56 females aged 60-84 years old, (71.43±8.67) years old. According to NYHA classification, there were 40, 91 and 23 Grade II, III and IV cases, respectively. Meanwhile, 9, 31, 24 and 78 cases were complicated with alcoholic heart disease, hypertensive cardiomyopathy, dilated cardiomyopathy and coronary artery disease, respectively. The 154 patients were randomly divided into an observation group and a control group (n=77), and their sex ratio, age, blood pressure, basic medication, and levels of fasting blood sugar (FBS), glycosylated hemoglobin and LDL-C were similar (P>0.05) (Table-I).

**Fig.1:**
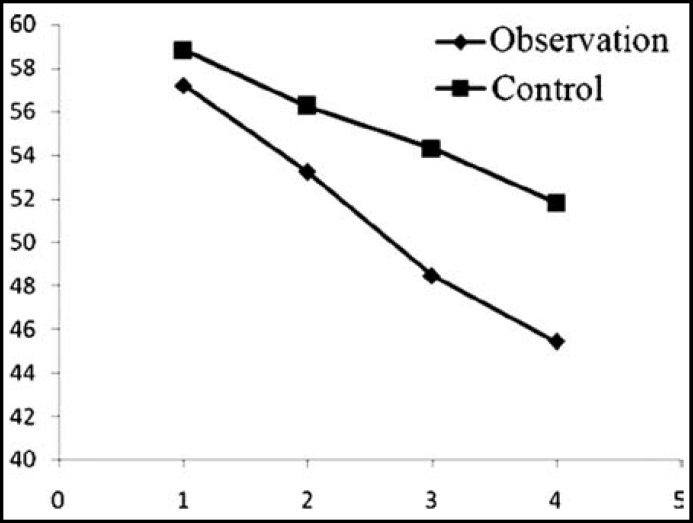
LVESD changes before treatment, and 1, 3 and 6 months after treatment

**Fig.2:**
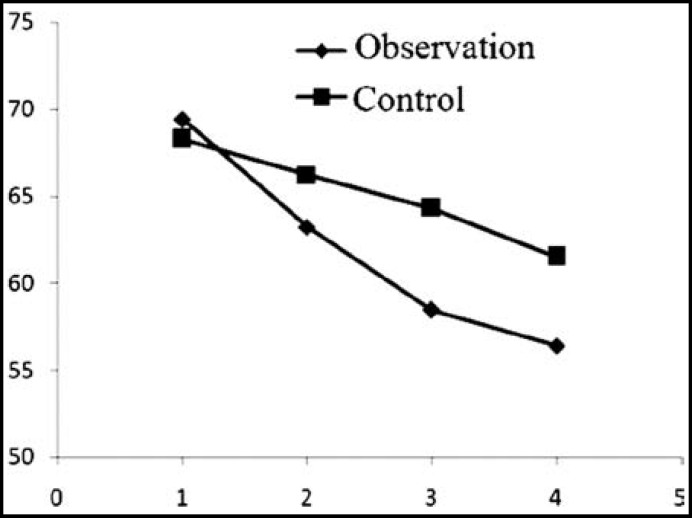
LVEDD changes before treatment, and 1, 3 and 6 months after treatment

**Fig.3:**
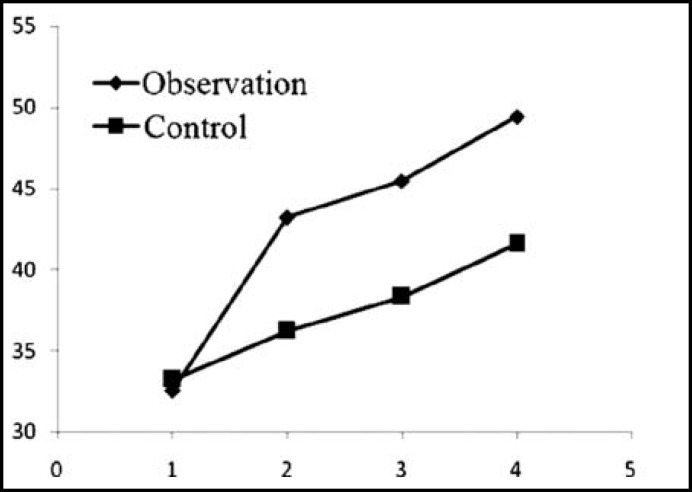
LVEF changes before treatment, and 1, 3 and 6 months after treatment

***Cardiac*** ***F*** ***unctions of the*** ***T*** ***wo*** ***G*** ***roups before and after*** ***T*** ***reatment: ***The NYHA classification, LVESD, LVEDD, LVEF and 6-minutes walking distance of the two groups after treatment were significantly better than those before (P<0.05). In the meantime, the indices of the observation group significantly exceeded those of the control group after treatment (P<0.05) (Table-II, Fig. 1-3).

***Incidence*** ***R*** ***ates of*** ***C*** ***ardiac*** ***E*** ***vents in the*** ***T*** ***wo*** ***G*** ***roups: ***During 6 months of follow-up, there were no sudden deaths in the observation group, with one case of cardiac death and two cases of re-admission due to aggravated heart failure (incidence rate: 3.90%). In the control group, there were two cases of sudden death, three cases of cardiac death and 6 cases of re-admission owing to aggravated heart failure (incidence rate: 14.29%). The incidence rate of cardiac events in the observation group was significantly lower than that of the control group (P<0.05) (Table-III).

***Security and*** ***T*** ***olerance*** ***E*** ***valuations on the*** ***T*** ***wo*** ***G*** ***roups: ***The edema, atrioventricular block, levels of blood lipid and blood sugar, and hepatic and renal functions of the observation groups remained stable during treatment. The average FBS levels and glycosylated hemoglobin contents of the two groups did not differ significantly (P>0.05). During the follow-up, 19 out of the patients in the observation group were subjected to Degree I atrioventricular block, which, however, did not affect the escalation of metoprolol dose. In case of 50 mg metoprolol daily, the heart rates of 30 patients reduced to 55 bpm. Besides, 29 patients suffered from decreased systolic pressure to 90 mmHg after being administered with 90 mg metoprolol. Only 4 patients managed to tolerate 200 mg metoprolol. Overall, the average daily dose of metoprolol was 105.33 mg. Despite the lowered blood pressure and resting heart rate of the observation group compared with those before treatment, the results were still maintained normal.

## DISCUSSION

In this study, the NYHA classification, LVESD, LVEDD, LVEF and 6-minutes walking distances of the two groups were significantly improved after treatment compared with those before treatment. Therefore, their cardiac functions were remarkably improved. Meanwhile, the incidence of cardiac events in the observation group was significantly lower than those of the control group, suggesting the treatment exerted satisfactory preventive effects.

The slightly decreased blood pressure and heart rate of the observation group were acceptable, accompanied by moderate drug tolerance. In the meantime, this group experienced neither evidently varied levels blood lipid and blood sugar, and hepatic and renal functions before and after treatment, nor severe adverse reactions such as hypotensive shock, edema and high-degree atrioventricular block. The results suggest that target-dose metoprolol treated the patients safely.^16^

**Table-I:** General information of the two groups

*Group*	*Case No. (n)*	*Gender*		*Age* *(years old)*	*Blood pressure (mmHg)*		*FBS* *(mmol/L)*	*Glycosylated hemoglobin (%)*	*LDL-C* *(mmol/L)*
		*Male*	*Female*		*Systolic pressure (mmHg)*	*Diastolic pressure (mmHg)*			
Observation	77	50	27	71.46±7.91	137.43±22.5	79.2±6.91	6.55±1.39	6.62	3.29±0.98
Control	77	48	29	71.35±9.21	141.15±19.89	80.22±7.02	6.49±1.32	6.53	3.44±0.95

**Table-II:** Cardiac functions of the two groups before and after treatment

*Group*	*Detection time*	*NYHA classification*	*LVESD (mm)*	*LVEDD (mm)*	*LVEF (%)*	*6-min walking distance (m)*
Observation	Before	3.22±0.61	57.22±6.94	69.42±6.29	32.58±7.73	227.32±189.42
	After	2.21±0.39^▲^^△^	45.43±7.01^▲^^△^	56.42±6.32^▲^^△^	49.42±9.21^▲^^△^	436.42±201.56^▲^^△^
	Difference induced by treatment	0.92±0.04^△^	7.92±0.61^△^	10.02±0.37^△^	14.59±2.12^△^	187.43±17.49^△^
Control	Before	3.24±0.67	58.83±9.17	68.26±7.43	33.25±9.19	229.53±190.63
	After	2.58±0.42^▲^	51.82±9.22^▲^	61.53±7.06^▲^	41.58±8.57^▲^	347.46±178.52^▲^
	Difference induced by treatment	0.72±0.03	3.39±0.43	6.32±0.17	7.94±1.97	98.32±14.24

Compared with the group before treatment, ^▲^P<0.05; compared with the control group,^△^P<0.05.

**Table-III:** Incidence rates of cardiac events in the two groups

*Group*	*Sudden death*	*Cardiac death*	*Re-admission due to aggravated heart failure*	*Incidence rate (%)*
Observation	0	1	2	3.90
Control	2	3	6	14.29

However, the average daily dose (105.33 mg) was far lower than the daily dosage commonly used (200 mg),^17^ which may be associated with the hindered atrioventricular transmission and sinus node functions of the elderly patients. To augments medication compliance and to guarantee clinical therapeutic effects and medication security, it is imperative to monitor the alterations of blood lipid and blood sugar levels, and hepatic and renal functions in the midst of initial treatment and escalated medication phases. On the other hand, the outcomes provide valuable evidence for similar studies in the future. 

In summary, besides being highly safe and tolerable, target-dose metoprolol can remarkably improve the NYHA classification, LVESD, LVEDD, LVEF and 6-minutes walking distance of the chronic heart failure patients complicated with DM while lowering the incidence rates of cardiac events.

## Authors Contributions:

**LXY:** Designed the protocol and prepared the manuscript.

**ZCF, ZPT, ZZH and JN:** Clinical data collection and experiments.

**SSO and ZBL:** Data collection and analysis.

**SYM:** Manuscript preparation and revision.
